# A novel approach to create an antibacterial surface using titanium dioxide and a combination of dip-pen nanolithography and soft lithography

**DOI:** 10.1038/s41598-018-34198-w

**Published:** 2018-10-25

**Authors:** Santiago Arango-Santander, Alejandro Pelaez-Vargas, Sidónio C. Freitas, Claudia García

**Affiliations:** 1 GIOM Group, Faculty of Dentistry, Universidad Cooperativa de Colombia – Campus of Medellín, Medellín, Colombia; 2 Cerámicos y vítreos Group, School of Physics, Universidad Nacional de Colombia – Campus of Medellín, Medellín, Colombia

## Abstract

Soft lithography and Dip-Pen Nanolithography (DPN) are techniques that have been used to modify the surface of biomaterials. Modified surfaces play a role in reducing bacterial adhesion and biofilm formation. Also, titanium dioxide has been reported as an antibacterial substance due to its photocatalytic effect. This work aimed at creating patterns on model surfaces using DPN and soft lithography combined with titanium dioxide to create functional antibacterial micropatterned surfaces, which were tested against *Streptococcus mutans*. DPN was used to create a master pattern onto a model surface and microstamping was performed to duplicate and transfer such patterns to medical-grade stainless steel 316L using a suspension of TiO_2_. Modified SS316L plates were subjected to UVA black light as photocatalytic activator. Patterns were characterized by atomic force microscopy and biologically evaluated using *S. mutans*. A significant reduction of up to 60% in bacterial adhesion to TiO_2_ -coated and -micropatterned surfaces was observed. Moreover, both TiO_2_ surfaces reduced the viability of adhered bacteria after UV exposure. TiO_2_ micropatterned demonstrated a synergic effect between physical and chemical modification against *S. mutans*. This dual effect was enhanced by increasing TiO_2_ concentration. This novel approach may be a promising alternative to reduce bacterial adhesion to surfaces.

## Introduction

Bacterial adhesion to the surface of biomaterials is the first step in colonization and biofilm formation^1^. Once bacteria have colonized a surface and biofilm has formed, a clinical infection around the biomaterial is presented, which will lead to biomaterial failure or chronic infections^2^. Therefore, a number of alternative approaches have been proposed and investigated to reduce bacterial adhesion to surfaces, since traditional approaches, like antibiotic therapies, are not recommended as biofilms are highly resistant to this conventional therapy^3^. These approaches include immobilization or release of antibacterial substances such as silver^4^, creation of anti-adhesion surfaces^5^, and fabrication of structured arrays^6–8^.

A reduction in bacterial adhesion and biofilm formation has been demonstrated by several authors on a variety of modified substrates using different bacterial species^6–8^. Multiple techniques have been employed to fabricate arrays on the surface of materials, including direct methods like dip-pen nanolithography (DPN)^9,10^ and indirect techniques like microstamping (soft lithography)^11,12^. Most investigations analysing the effect of fabricated surfaces on bacterial adhesion and cellular behaviour share photolithography and soft lithography as the combination of choice to modify the surface of the tested material^13–16^. Soft lithography is a series of indirect techniques that allow the fabrication of micro and sub micro arrays on the surface of materials using an elastomeric stamp that is usually made of poly(dimethylsiloxane)^11,17,18^. This PDMS stamp is obtained from a master model, which is usually fabricated using photolithography^13,15^. However, photolithography presents a series of disadvantages, including high cost, lack of chemical control of the surface, and inapplicability over non-planar surfaces, which render it difficult to use, especially in the biomedical field^19^. DPN has some distinct advantages, including the fact that it is a direct-writing technique that is compatible with many substrates and inks and shows high resolution and registration^10^. It has been proposed that these physical modifications act as barriers or obstacles that hinder the interaction of a single bacterium or a cluster of bacteria with other bacteria as well delaying the cell-to-cell communication, known as quorum-sensing^20^, which is recognized to play an important role in biofilm formation by bacterial species^21,22^.

On the other hand, some chemical substances are recognized to be antibacterial. Metals like silver in different forms (silver nitride and silver nanoparticles) have been long recognized as antibacterial substances^23,24^, as well as copper and zinc^25^. However, these metals present some drawbacks, including cytotoxicity, inflammatory responses and increase in bacterial resistance^26^. Other compounds, such as titanium dioxide (TiO_2_), have long been studied for their antibacterial properties. TiO_2_ has received special attention as an antibacterial material, particularly in relation to the photocatalytic effect^27–29^.

This photocatalytic effect is produced when reactive oxygen species (ROS), such as hydroxyl radicals, hydrogen peroxide, and superoxide anion, are generated after TiO_2_ is exposed to UV light. Such ROS are known to inactivate bacteria, viruses, and fungi^30^. The basic principle of photocatalysis consists on the formation of an electron-hole pair upon absorption of a photon with an energy equal or higher than the semiconductor’s band-gap^31^. Two reactions then occur simultaneously: oxidation from photogenerated holes and reduction from photogenerated electrons^32^. After these electron-hole pairs in the semiconductor particles are formed, the electron migrates to the metal and becomes trapped, therefore electron-hole recombination is inhibited^33^. The photogenerated hole is transferred to the target molecule (usually an organic compound) causing its oxidation or destruction in the case of bacteria or viruses^31^. TiO_2_ has been considered as an ideal photocatalyst due to the fact that it is inexpensive, chemically highly stable, and the photogenerated holes are highly oxidizing^29^. Even though TiO_2_ exists in three crystalline forms (anatase, rutile, and brookite), the anatase form has shown to have the highest photoactivity^34^. This effect has been largely proposed for disinfection of polluted water^30,35,36^, although it has also been proposed in the biomedical field due to promising *in vitro* results in reducing bacterial adhesion to biomaterials^37,38^.

Consequently, the combination of a physical surface modification approach with the use of an antibacterial substance to further increase bacterial reduction needs to be addressed. Therefore, the main objective of this work was to assess whether a physical surface modification approach using TiO_2_ as antibacterial compound subjected to UV light might reduce *Streptococcus mutans* adhesion to surgical-grade stainless steel plates.

## Materials and Methods

### Substrates

For dip-pen nanolithography (DPN), commercial 10 mm × 10 mm × 1 mm gold wafers (Nanoink Inc., USA) were used as model substrates due to their affinity for the ink.

For soft lithography (microstamping), stainless steel 316L plates (SS316L) 10 mm × 10 mm × 1 mm plates (Onlinemetals, USA) were polished up to 1 µm diamond paste (Leco Corporation, USA) to obtain a mirror-like surface. SS316L plates were sequentially cleaned using surfactant, acetone (99.8% v/v, Merck Millipore, USA), distilled water and absolute ethanol (99% v/v, Merck Millipore, USA) for 8 min each in an ultrasound bath and let dry in air. For simplicity, this surface will be referred as SS polished through the text and used as control.

### Chemicals

For DPN, a commercial polymeric adhesive (Norland Optical Adhesive 68 T, Norland Products Inc, USA) was used. It was kept at 4 °C throughout the experiments to maintain viscosity.

For microstamping, a silica sol was prepared using the one-stage sol-gel method as previously described^39,40^. Tetraethylorthosilicate (TEOS) and methyltriethoxysilane (MTES) (ABCR GmbH & Co., Germany) were used as silica precursors for the hybrid sol, 0.1 N nitric acid (Merck Millipore, USA) and acetic acid (glacial, 100% v/v, Merck Millipore, USA) as catalysts and absolute ethanol (99.9% v/v, Merck Millipore, USA) as solvent. The final concentration of SiO_2_ was 18 gL-1. Commercial titanium oxide (TiO_2_) anatase nanoparticles (<100 nm particle size, NaBond, Hong Kong) were added to the silica sol at 5 and 10% concentrations by weight and the suspension was agitated to reach homogeneity. It was kept at 4 °C.

### Surface preparation

**Figure 1:**
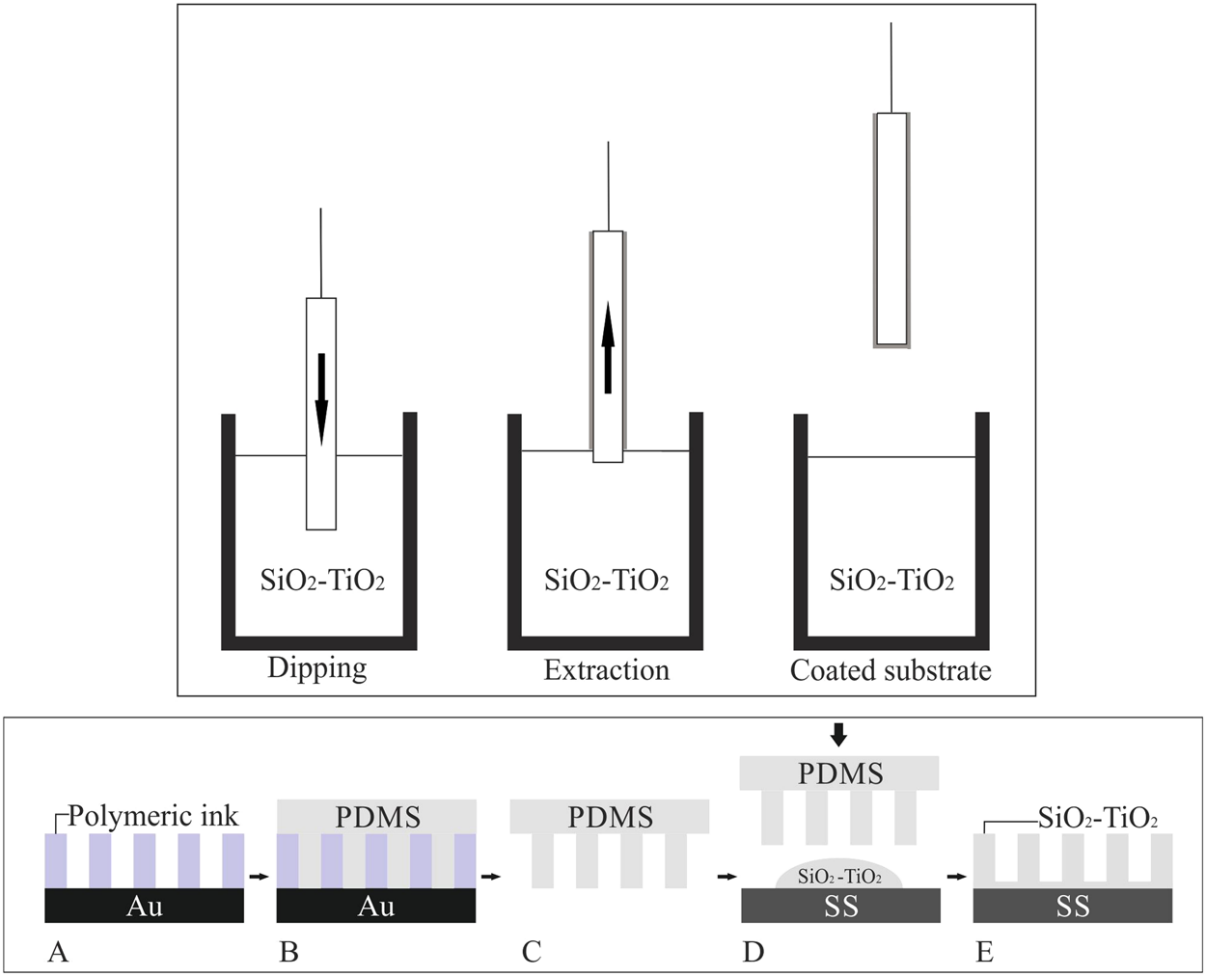
Top: dip coating of SS316L using SiO_2_-TiO_2_ suspension. Bottom: patterning process using microstamping. Pattern fabrication on gold (**A**), PDMS pre-polymer poured onto pattern (**B**), PDMS stamp after polymerization (**C**), microstamping using SiO_2_-TiO_2_ suspension (**D**) and transferred SiO_2_-TiO_2_ pattern on SS316L.

Three types of samples were prepared: a control group with SS polished as already described and two experimental groups: SS-TiO_2_ coated prepared by dip-coating with a SiO_2_-TiO_2_ suspension and SS-TiO_2_ micropatterned obtained by combination of dip-pen nanolithography and soft lithography (Fig. 1).

#### Preparation of SS-TiO2 coated surfaces

SS316L plates were coated following the dip-coating method. The previously cleaned SS316L plates were dipped in SiO_2_-TiO_2_ suspension with 5% or 10% TiO_2_ concentration and released at a speed of 4 cm/min with the purpose of immersing and coating the metallic plate completely in the suspension. The coating was allowed to dry for 10 min in air and then the coated plates were subjected to heat treatment in a furnace following the next protocol: temperature was raised 5 °C per minute up to 100 °C; this temperature was kept for 30 minutes to continue at 5 °C per minute up to 200 °C for 30 min. The final step was to raise it 5 °C per minute up to 400 °C and this temperature was kept for 30 min. This final temperature was established to be distant from anatase-to-rutile transformation temperatures as they start in the range of 500 to 600 °C^41^. These surfaces are referred as SS 5% TiO_2_ coated and SS 10% TiO_2_ coated throughout the text.

#### Preparation of SS-TiO_2_ micropatterned surfaces

Column Pattern master fabrication:DPN was carried out as previously described by us^42^. Briefly, a NLP 2000 system (NanoInk Inc., USA) was used and 0.4 µL of polymeric ink were injected into each well of a twelve-well plate (NanoInk Inc., USA). M triangular tips (10-tip arrays) were selected to deposit the ink on the substrates to create a master designed (~ 10 mm^2^) column array disposed in an 11 × 11 matrix.

Soft lithography (Microstamping):Polydimethylsiloxane (PDMS) (Silastic T-2, Dow Corning Corporation, USA) was used to duplicate the master created on gold. PDMS was prepared according to the manufacturer and cured for 24 h. Then, it was carefully removed from the surface and thermally treated at 80 °C for 3 h to complete polymerization. The PDMS was used as a microstamp to transfer the SiO_2_-TiO_2_ suspension to SS316L surfaces. 7 µL of the suspension with 5% or 10% TiO_2_ concentration were deposited onto the SS316L surface, a PDMS microstamp was placed over the drop, applying gentle digit pressure, and the suspension was allowed to gel for 4 h at RT. The PDMS stamp was then carefully removed and the SS316L plate with the transferred pattern was heat treated following the same protocol as the coating. These surfaces are referred as SS 5% TiO_2_ micropatterned and SS 10% TiO_2_ micropatterned throughout the text.

### Surface characterization

Column master, SS, SS-TiO_2_ coated and SS-TiO_2_ micropatterned plates were characterized using atomic force microscopy (AFM) (Nanosurf Easyscan 2, Nanosurf AG, Switzerland) in tapping mode performed with a NCLR (Nanosensors™, Switzerland) tip at a force constant of 48 N/m. Images post processing was performed using software AxioVision (V 4.9.1.0, Carl Zeiss Microscopy GmbH, Germany), software Image J 1.51 J^43^, and software WSxM 5.0^44^.

SS316L surface properties were evaluated by AFM as described above and by contact angle measurement using distilled water. AFM images of 50 µm × 50 µm were used for surface roughness measurements with the arithmetic average of the roughness profile (Ra) calculated using software for AFM analysis (Gwyddion 2.34, Department of Nanometrology, Czech Metrology Institute, Czech Republic). Contact angle measurements followed the sessile drop method on 10 random plates from each group using a camera (Canon EOS Rebel XS, Japan) and a macro lens (105 mm F2.8 EX DG OS, Sigma, USA) with the angle values obtained using software Axio Vision. In addition, Energy-Dispersive X-Ray Spectroscopy (EDX) was used to chemically characterize SS polished and SS-TiO2 coated surfaces using a scanning electron microscopy (JEOL JSM-5910LV, Japan).

### Biological characterization

*Streptococcus mutans* was used as model cariogenic bacteria to assess the antibacterial properties of developed surfaces. *S. mutans* Clarke ATCC 25175 (Microbiologics, USA) was seeded in brain heart infusion (BHI) agar (Scharlab S.L., Spain) supplemented with 0.2 U/ml bacitracin (Sigma Fluka, USA) and grown at 37 °C for 18–24 h. Then, they were cultured in peptone water broth medium (3% peptone and 20% sucrose) at 37 °C for 16 h. Afterwards, the bacterial culture was centrifuged at 5000 g for 15 min, the supernatant was discarded and the bacterial pellet was re-suspended in peptone medium at 10^7^ CFU/mL by measuring the nephelometric turbidity unit (NTU) (based on a calibration curve of NTU vs CFU/mL). 1 ml of bacterial solution was added to each well of a 24-well non-treated polystyrene plate (Costar, Corning Inc., USA) containing the surfaces, which were previously sterilized with 70% alcohol. The plate was incubated at 37 °C for 8 h to allow bacterial adhesion to control and experimental surfaces. Then, a set of samples of the different surfaces was exposed to UVA black light (EIKO F8T5/BL, 8 W, 350 nm peak emission) for 60 min as it has been reported by other authors^38,45^ and the other sample set was not subjected to this treatment. After the 60 min, samples were removed, washed three times with 500 µl of 0.9% saline solution to remove non-adherent bacteria and prepared for characterization methods.

For the quantification of viable adherent bacteria, surfaces were subjected to a 3-second sonication at 50% power (Qsonica 125, 1/8″ probe tip, USA) in 10 ml of 0.9% saline solution, serial dilutions performed and 10 µL from dilution were cultured in BHI agar in triplicate following the drop plate method^46^. Culture plates were incubated at 37 °C for 48 h and then colony forming units (CFU) were counted.

For analyses of bacterial adhesion morphology and coverage by scanning electron microscopy (JEOL JSM-5910LV, Japan), surfaces were incubated with 3% glutaraldehyde to permanently fix bacterial cells. SEM images at 2000X and 5000X magnifications were randomly taken from the center and the periphery of each sample.

This entire process was repeated in three independent assays.

### Statistical analysis

Experimental results are presented as the mean ± standard deviation (SD). Comparison between conditions was performed using the one-way ANOVA test with post-hoc Tukey method. Values of p < 0.05 were considered statistically significant. Software SPSS (V. 22) was used for statistical analysis.

The datasets generated during and/or analysed during the current study are available from the corresponding author on reasonable request.

## Results

### Master fabrication

**Figure 2:**
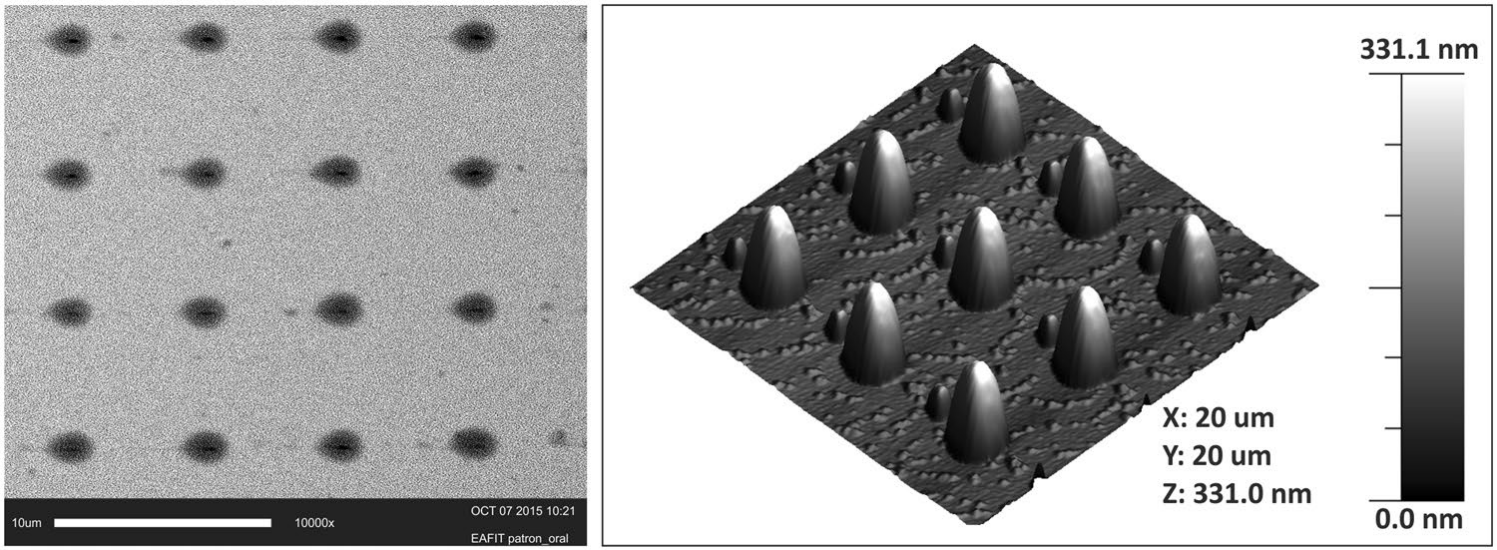
SEM (left) and AFM (right) images of polymeric column pattern on gold.

A commercially available polymeric adhesive was used to fabricate patterns on a model gold substrate. A column pattern was created and the dimensions were determined using the DPN system and confirmed by AFM and SEM. Column averaged 2.9 ± 0.1 µm in diameter and 250 to 450 nm in height. Figure 2 shows SEM and AFM images of a pattern fabricated on gold. As size variability was observed among features in different patterns, care was taken to select those approaching a size near the *S. mutans* and its arrangement as the bacterial species evaluated. Satellite columns were observed next to the main columns. However, these satellite columns showed sizes that were five to six times smaller than the main columns, which is negligible for the size of the bacterial species used in the current investigation. Furthermore, these satellite columns did not seem to affect the PDMS nor the micro stamping process.

### Microstamping

**Figure 3:**
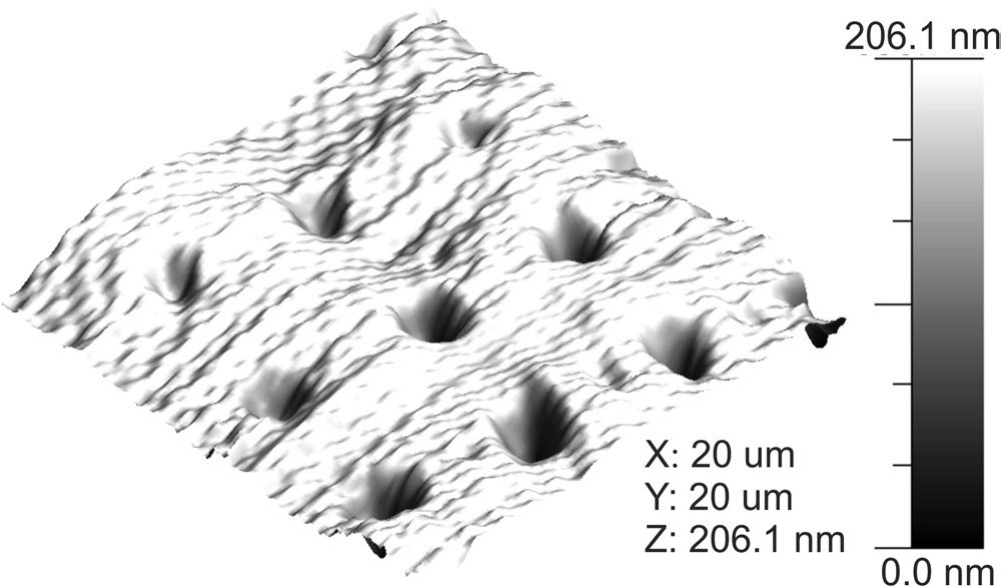
AFM image of PDMS stamp.

In Fig. 3, a 20 × 20 µm AFM image of the PDMS stamp shows that the process was successful. The PDMS was able to reproduce the original features in the same height range (200–500 nm) and diameter (∼2.5 µm) as the original pattern.

### Surface properties

**Figure 4:**
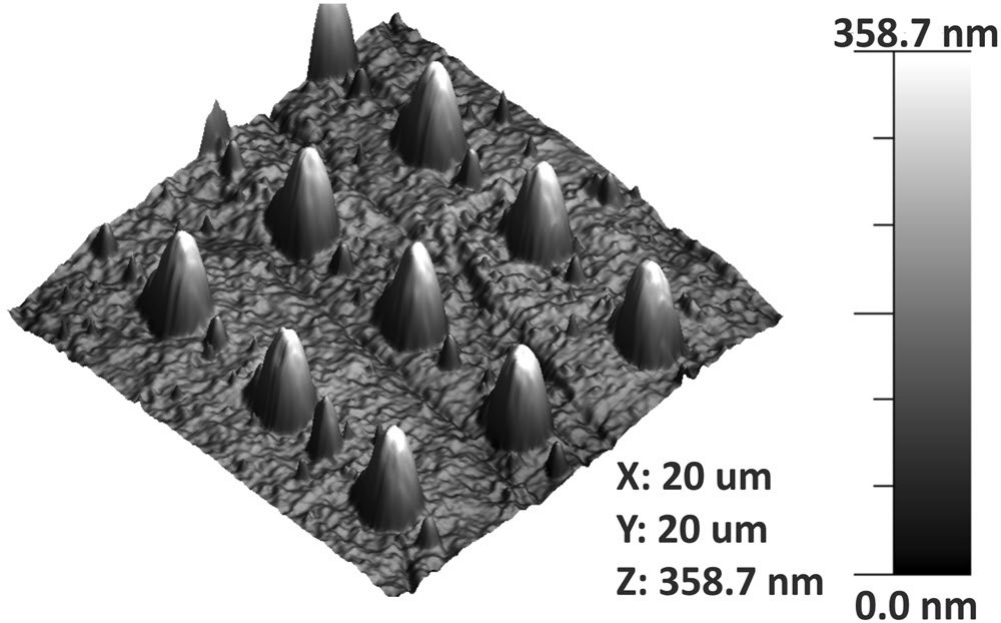
AFM image of TiO_2_ pattern after transferring to SS316L.

A suspension of TiO_2_ nanoparticles was used to transfer the arrays fabricated on gold to SS316L. Transfer of column patterns was successful, and features were conserved in the same size range (250–450 nm) as the features created on the original substrates (Fig. 4).

**Figure 5:**
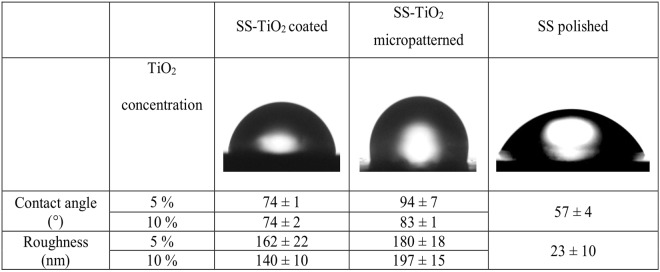
Water contact angles images of SS-TiO_2_ coated, SS-TiO_2_ micropatterned, and SS polished and contact angle and roughness average (Ra) measurements.

Contact angle measurements (Fig. 5) showed a statistically significant increase from SS polished to SS-TiO_2_ coated surfaces at both TiO_2_ concentrations and from these to SS-TiO_2_ micropatterned especially at a 5% TiO_2_ concentration (p = 0.001). There was no difference between SS-TiO_2_ coated at both concentrations while a statistically significant difference occurred between the SS-TiO_2_ micropatterned surfaces at 5% and 10% TiO_2_ concentration (p = 0.001).

Regarding roughness, values also increased from SS polished to SS-TiO_2_ coated surfaces and to SS-TiO_2_ micropatterned at both TiO_2_ concentrations (Fig. 5). The difference in roughness was statistically significant between SS polished and all other surfaces, SS 10% TiO_2_ coated and micropatterned surfaces at both TiO_2_ concentrations, and SS 10% TiO_2_ micropatterned and coated surfaces at both TiO_2_ concentrations (p = 0.001). Interestingly, no difference occurred between surfaces (coated and micropatterned) with the lowest concentration of TiO_2_.

**Figure 6:**
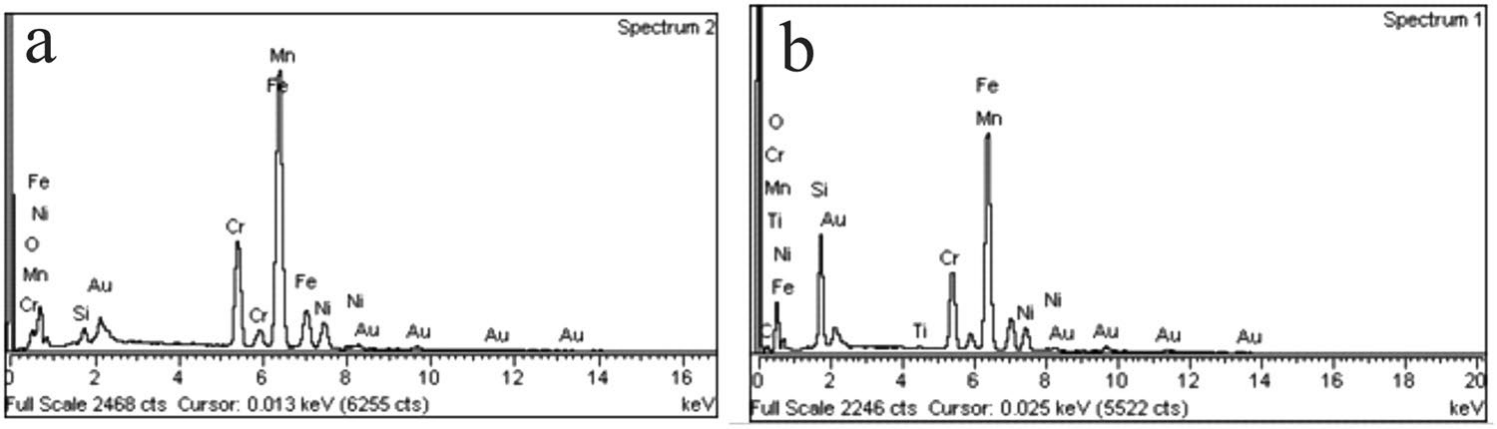
EDS spectrum of the SS polished (**a**) and SS-TiO_2_ coated (**b**) surfaces.

EDS was used to chemically characterize the surfaces. Figure 6 shows the spectrum of the SS polished and SS- TiO_2_ coated where the presence of titanium was observed.

### Biological characterization

**Figure 7:**
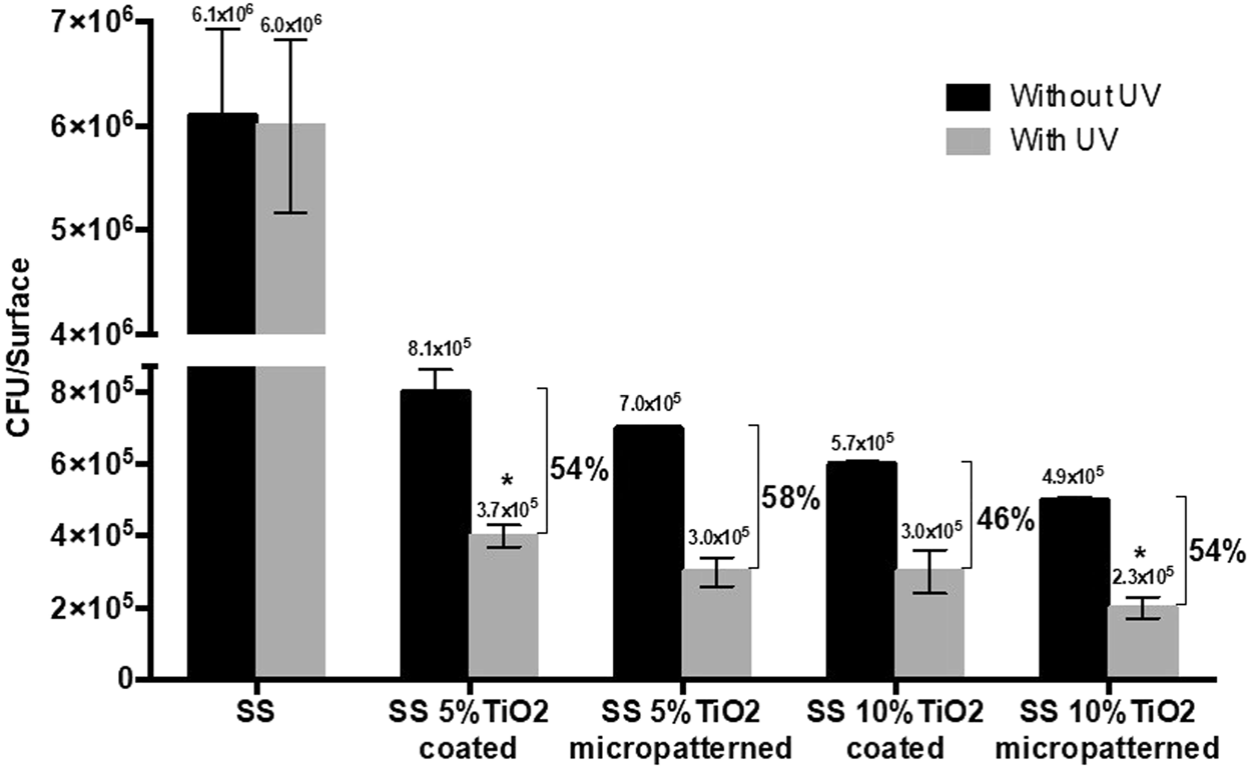
Viable adhered bacteria on SS-TiO_2_ coated and SS-TiO_2_ micropatterned with different TiO_2_ concentration after exposure to UV light or not. Percentages indicate the decrease of viable adhered bacteria due to UV exposure. All samples without UV are statistically significantly different between them. *Statistically significant difference compared to all other conditions.

**Figure 8:**
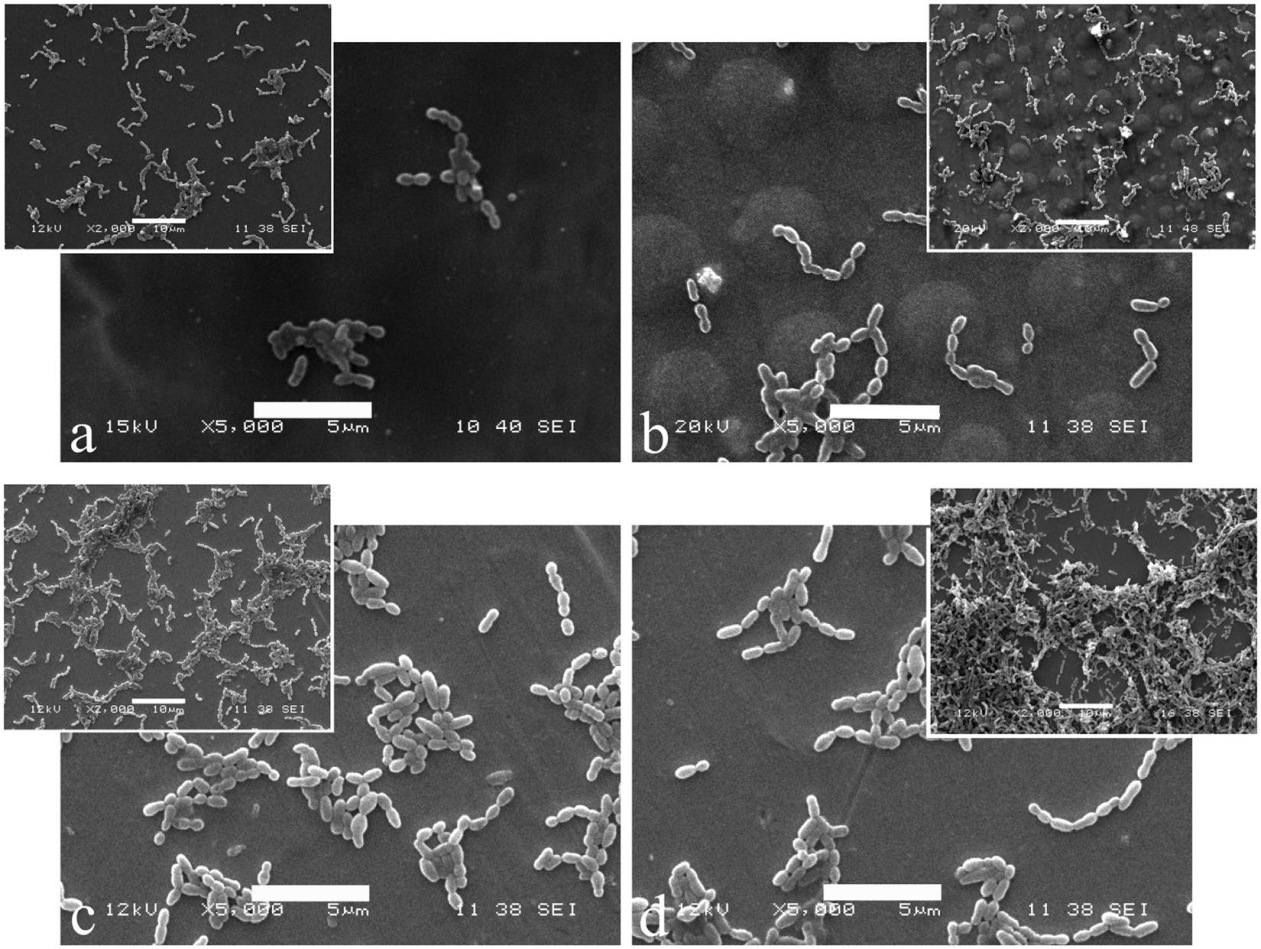
SEM photographs of bacterial colonization on SS-TiO_2_ coated (**a**), SS-TiO_2_ micropatterned (**b**), SS polished (**c**) and unexposed SS polished (**d**). Scale is 5 µm. Inserts show interaction at 2000 × . Scale in the inserts is 10 µm.

*Streptococcus mutans* was incubated with the three substrates (SS polished, SS-TiO_2_ coated, and SS-TiO_2_ micropatterned) to allow their adhesion to the surfaces for 8 h before being exposed to UVA black light for 60 min. Preliminary assays showed and confirmed that, regardless of surface type, the adherent bacteria remains viable in the same amount immediately before and after 60 min in saline solution, meaning that there is neither bacterial growth nor death during this period of time under this incubation condition. SS polished showed an adhesion of 6.1 × 10^6^ CFU/surface, which is the highest bacterial adhesion, more than one order of magnitude that the other surfaces with or without exposure to UV light (Fig. 7). Surfaces with UV exposure showed lower number of viable adhered bacteria than the ones without this treatment with a ∼50% decrease of cell viability among the different surfaces, demonstrating the photocatalytic effect against the adhered bacteria. Moreover, Fig. 7 also shows that patterning the SS316L plates with TiO_2_ particles at both 5 and 10% concentrations exhibited lower adhesion than the corresponding coated surfaces with or without UV exposure, which could be associated to the physical modification. Interestingly, under exposure to UV light, the SS micropatterned with 5% TiO_2_ showed similar viable adhered bacteria than the SS coated with 10% TiO_2,_ indicating a synergic effect of physical and chemical modification against the bacteria. Finally, SS surfaces micropatterned with the highest TiO_2_ concentration (10%) showed the lowest viable adhered bacteria, which corresponds to a 96% reduction in bacterial adhesion to stainless steel. SEM images confirmed these results as lower numbers of bacteria were observed on the micropatterned TiO_2_ surfaces compared with the coated TiO_2_ surfaces and exposed and unexposed SS polished (Fig. 8).

## Discussion

Several techniques have been used to pattern the surface of materials, including direct methods like photolithography and dip-pen nanolithography (DPN)^9,13,16^ and indirect techniques like soft lithography^11,12^. Micro arrays on the surface can be fabricated using soft lithography, which uses an elastomeric stamp that is usually made of poly (dimethyl siloxane) (PDMS)^11,17,18^. This stamp is a negative from a master model that is usually fabricated using photolithography^13,15^. However, high costs, difficulty to control the chemistry of the surface and difficulty to be used by biologists and related personnel make photolithography difficult to use, especially in the biomedical field^19^.

In the present work, DPN was used to manufacture a master model and silica sol modified with TiO_2_ nanoparticles was used to transfer the pattern to SS316L, to produce an array of an antibacterial substance on a surgical-grade SS surface. This novel approach aimed to analyse whether a physical modification complemented with an antibacterial substance might show a synergic effect for reduction of bacterial adhesion.

A column pattern was created on model surfaces using DPN. The diameter of the features in the current work are in the order of microns (1–5 µm) and the height in the range of 150 to over 400 nm, although DPN allows the creation of features in smaller, nanometric sizes (below 100 nm)^10^. Hochbaum and Aizenberg^7^ proposed that the size of the fabricated features should be similar to that of the tested bacterial species in order to reduce bacterial adhesion. Since *S. mutans* organise in short chains of three or four bacterial cells, column height, pitch, and separation between two consecutive columns was designed considering the fact that these physical obstacles should surround these chains of bacteria, rather than one single bacterial cell.

The transfer process with soft lithography allowed the dimensions of the fabricated patterns on gold to be preserved on stainless steel as PDMS is capable of copying features smaller than those created in this work^47^. In addition, surface characteristics were also evaluated by measuring roughness and contact angle. Coating and patterning the SS316L with TiO_2_ showed ∼10 times higher roughness than SS coated with only SiO_2_ (data not shown), which may be explained by the fact that the size of TiO_2_ nano particles may render the suspension coarser and, once deposited, increased the roughness values. Several authors have positively correlated roughness and adhesion of *S. mutans* to different biomaterials^48–50^ and both TiO_2_ coated and micropatterned surfaces showed higher roughness than SS polished; therefore, the lower number of bacteria found on both experimental surfaces (TiO_2_ coated and micropatterned) may be associated with the antibacterial effect of TiO_2_ and with the obstacle caused by the patterns in the case of the TiO_2_ micropatterned surface rather than surface roughness. Regarding hydrophobicity, there was an increase after coating or patterning the SS surface with the TiO_2_ suspension. As the differences in roughness and wettability between modified surfaces is insignificant, the antibacterial effect observed on both surfaces may be better explained by the presence of TiO_2_.

Bacterial adhesion to a biomaterial is a process that involves many variables, including surface properties, physico-chemical characteristics of bacteria and environmental factors^51^, all of which play a key role during the early steps of adhesion and biofilm formation. Regarding the disruption of bacterial adhesion and biofilm formation, Ready *et al*.^52^ suggested that the efficacy of antibacterial substances may be observed after adhesion as the biofilm matures. In the current work, bacterial adhesion to TiO_2_ micropatterned surfaces showed a reduction between 50 and 60% after exposure to UVA light compared with unexposed surfaces regardless of TiO_2_ concentration. TiO_2_ micropatterned presented lower viable adhered bacteria than coated surfaces for each TiO_2_ concentration, which indicates that reduction of bacterial adhesion was achieved not only by the photocatalytic effect of TiO_2_, but also by the physical obstacles that further reduced adhesion. The available contact area is relevant for bacteria to adhere to a surface and, since *S. mutans* organise in short chains, the pattern created on the surface may have acted as physical obstacles for such chains to organise, expand, and find each other (quorum sensing) as proposed by Hochbaum and Aizenberg^7^. SEM images (Fig. 8) showed those physical obstacles created by patterning the SS surface with TiO_2_ while coated surface is smooth, as indicated by AFM measurements, allowing the contact between bacteria cells and a higher adhesion. Complementary, the photocatalytic effect assisted in further reducing bacterial adhesion to the TiO_2_ coated and TiO_2_ micropatterned, as shown by the 10% TiO_2_ micropatterned surface with the lowest viable adhered bacteria after UV exposure and even more by SS micropatterned with 5% TiO_2_ having similar viable adhered bacteria than coated with 10% TiO_2_.

Furthermore, as mentioned before, the most hydrophobic surface showed the lowest bacterial adhesion, which may be explained by the fact that this bacterial strain possesses a more hydrophilic surface. Satou *et al*.^53^ found that different strains of *S. mutans* have a hydrophilic surface and that hydrophilic bacterial show higher adhesion to hydrophilic surfaces.

In regarding to other works, the results here presented are in accordance with other authors by showing that the presence of micropatterns reduces the adhesion of *S. mutans* by 96%. These values are higher than the number reported by Chung *et al*.^6^, with 87% reduction in *S. aureus*, and similar to May *et al*.^8^, who obtained 95.6 to 99.9% reduction since they evaluated different bacterial strains.

These results also confirmed that TiO_2_ has a chemical antibacterial effect regardless of nanoparticle concentration and exposure time. Other authors have found that the exposure of different bacterial species to UV-photoactivated TiO_2_ results in a significant reduction of bacterial adhesion and biofilm formation. Chun *et al*.^45^ coated SS orthodontic wires with TiO_2_ to assess bacterial adhesion of *S. mutans* and found a significant reduction in bacterial adhesion between coated and non-coated wires (100 CFU vs 720 CFU, respectively). Choi *et al*.^38^ assessed bacterial adhesion to TiO_2_ and TiAg surfaces coated with TiO_2_ after exposure to UV light and found a significant reduction in *S. mutans* adhesion to treated surfaces. In addition, they found that the anatase form of TiO_2_ presented higher antibacterial activity than rutile. Even though the results of the current work demonstrated a reduction in bacterial adhesion after exposure of TiO_2_ coated and micropatterned surfaces, the values were not as high as the numbers reported by Chun *et al*. and Choi *et al*. However, Erdural *et al*.^54^ proposed that a high concentration of SiO_2_ in a SiO_2_-TiO_2_ suspension could lead to a reduced photocatalytic effect due to an excessive dilution of TiO_2_ in the SiO_2_, which will eventually lead to a reduced accessibility of photons and reduced transport of ROS between TiO_2_ and the bacterial suspension. They found the best antibacterial effect on *E. coli* when the TiO_2_ concentration was 8% wt, which is in agreement with the results of the current work where the TiO_2_ concentration was between 5 and 10% wt.

## Conclusions

Micropatterned surfaces with antibacterial TiO_2_ nanoparticles were successfully created on SS316L by a combination of dip-pen nanolithography and soft lithography. Those surfaces were able to reduce the viability of adhered bacteria at low TiO_2_ concentration (5%) in a similar degree than a simple coated surface with a higher TiO_2_ concentration (10%), showing a synergic effect between physical and chemical modification against *S. mutans*. This dual effect was further enhanced by increasing TiO_2_ in micropatterned surfaces to 10%. The results of this investigation showed that a combination of a physical and chemical approach is a promising alternative to reduce adhesion of viable bacteria to biomaterials.
